# Adjusting ventilator settings to relieve dyspnoea modifies brain activity in critically ill patients: an electroencephalogram pilot study

**DOI:** 10.1038/s41598-019-53152-y

**Published:** 2019-11-12

**Authors:** Mathieu Raux, Xavier Navarro-Sune, Nicolas Wattiez, Felix Kindler, Marine Le Corre, Maxens Decavele, Suela Demiri, Alexandre Demoule, Mario Chavez, Thomas Similowski

**Affiliations:** 1 Sorbonne Université, INSERM UMRS1158 Neurophysiologie Respiratoire Expérimentale et Clinique, F-75005 Paris, France; 2 AP-HP, Groupe Hospitalier Pitié Salpêtrière - Charles Foix, Département d’Anesthésie-Réanimation, F-75013 Paris, France; 3 Sorbonne Université, INSERM UMR 1127, CNRS UMR 7225, Institut du Cerveau et de la Moelle Épinière, Paris, France; 4 AP-HP, Groupe Hospitalier Pitié Salpêtrière - Charles Foix, Service de Pneumologie, Médecine Intensive et Réanimation, Département R3S, F-75013 Paris, France

**Keywords:** Diagnosis, Medical research

## Abstract

Dyspnoea is frequent and distressing in patients receiving mechanical ventilation, but it is often not properly evaluated by caregivers. Electroencephalographic signatures of dyspnoea have been identified experimentally in healthy subjects. We hypothesized that adjusting ventilator settings to relieve dyspnoea in MV patients would induce EEG changes. This was a first-of-its-kind observational study in a convenience population of 12 dyspnoeic, mechanically ventilated patients for whom a decision to adjust the ventilator settings was taken by the physician in charge (adjustments of pressure support, slope, or trigger). Pre- and post-ventilator adjustment electroencephalogram recordings were processed using covariance matrix statistical classifiers and pre-inspiratory potentials. The pre-ventilator adjustment median dyspnoea visual analogue scale was 3.0 (interquartile range: 2.5–4.0; minimum-maximum: 1–5) and decreased by (median) 3.0 post-ventilator adjustment. Statistical classifiers adequately detected electroencephalographic changes in 8 cases (area under the curve ≥0.7). Previously present pre-inspiratory potentials disappeared in 7 cases post-ventilator adjustment. Dyspnoea improvement was consistent with electroencephalographic changes in 9 cases. Adjusting ventilator settings to relieve dyspnoea produced detectable changes in brain activity. This paves the way for studies aimed at determining whether monitoring respiratory-related electroencephalographic activity can improve outcomes in critically ill patients under mechanical ventilation.

## Introduction

Dyspnoea is common in patients receiving mechanical ventilation (MV)^1^ despite the presence of ventilatory support. Dyspnoea under MV is highly anxiogenic and associated with negative clinical outcomes^1^. It has therefore been identified as one of the symptoms that should be assessed systematically in intensive care unit (ICU) patients^2^. Improving the management of dyspnoea is a clinical priority^3^ and can often be relieved very simply, for example by adjusting ventilator settings for patients with dyspnoea under MV^1^.

Dyspnoea under MV is often not recognized^4^ because the permanent presence of a caregiver at the bedside of the patient is generally not possible and because ICU physicians and caregivers do not perform well in diagnosing and evaluating dyspnoea in ICU patients with impaired self-reporting abilities^5–7^. This risk of ‘occult respiratory suffering’ justifies the need for surrogate diagnostic tools, including the respiratory distress observation scale (RDOS)^8^ and variants that have been optimised for the ICU context^9,10^. These clinical scales include a degree of subjectivity and their use requires the presence of a caregiver at the bedside. As such, they cannot provide an instantaneous detection of frequent acute dyspnoegenic events during MV. Neuromarkers of dyspnoea derived from a continuously recorded electroencephalogram (EEG) would address both the issue of subjectivity and the need for a permanent bedside presence. Electroencephalographic signatures of the reaction of the brain to experimental respiratory loading in healthy human subjects have been associated with breathing discomfort^11–13^ and have been observed in both an experimental model of dyspnoea under MV^11^ and in patients suffering from severe chronic respiratory insufficiency due to amyotrophic lateral sclerosis^12^. In both cases, parallelism was found between the adequacy of ventilatory support, respiratory discomfort, and respiratory-related cortical activity. In the above studies, the dyspnoea generating stimulus was either an added inspiratory or expiratory threshold load or a situation of respiratory muscle load-capacity imbalance. This tends to generate dyspnoea of the ‘excessive effort’ type and a cortical response that involves an important motor component. Dyspnoea of the ‘air hunger’ type, which is highly prevalent in mechanically ventilated patients^1^, stems from different mechanisms. It often involves the combination of hypercapnia and an insufficient ventilatory response, and mostly activates non-motor brain areas^14,15^. Electroencephalographic signatures have also been described in hypercapnia-induced breathlessness^16^, albeit different from the patterns observed in response to loading. All these observations lend support to the hypothesis that EEG data could be useful to identify and manage dyspnoea in ICU patients receiving MV by prompting caregiver interventions.

All of the above EEG studies have relied on event-related EEG processing strategies to identify respiratory-related cortical activation under the form of pre-inspiratory potentials (PIPs), probably originating in the supplementary motor area^11^. This approach involves averaging a large number of respiratory-synchronized EEG segments and is very sensitive to artefacts (e.g. due to patient movement or electronic interference). Alternatively, changes in brain activity and connectivity induced by inspiratory loading can accurately be detected using a computational method relying on the continuous analysis of EEG covariance matrices^17,18^. This approach is robust to interference and has the capacity for real-time detection^19^.

We therefore designed the present study to test the hypothesis that improving respiratory comfort by adjusting suspected inadequate ventilator settings in patients self-reporting dyspnoea under MV (or, for non-communicative patients, considered as being dyspnoeic according to the RDOS) would result in detectable cortical changes. We predicted that adjusting ventilator settings would be associated with clinical improvement in terms of dyspnoea and that either PIPs or a covariance-based EEG classifier, or both, would allow us to adequately distinguish the corresponding ‘before’ and ‘after’ conditions. To test this hypothesis, we conducted a first-of-its-kind pilot study in invasively mechanically ventilated patients.

## Materials and Methods

### Setting and patients

This observational study was conducted in the medical intensive care unit of the department of respiratory and intensive care medicine (16 beds) and in the surgical ICU of the department of anaesthesiology and intensive care (12 beds) of the Pitié Salpêtrière Hospital in Paris, France (tertiary university hospital, 1750 beds). The study was approved by the appropriate legal and ethical authority (*Comité de Protection des Personnes Ile-de-France VI Pitié-Salpêtrière*) that waived the need for written consent given the observational nature of the study and was conducted in agreement with the principles of the declaration of Helsinki.

Inclusion criteria were: (1) age >18 years; (2) MV through an endotracheal tube in pressure support mode for >24 hours; (3) absence of organ failure other than respiratory; (4) Ramsay scale 2 to 4^20^; (5) in Ramsay 2 or Ramsay 3 patients, a positive answer to the question ‘do you experience breathing difficulties’ and either a breathing frequency >20 breaths/min or visible inspiratory contractions of neck muscles; (6) in Ramsay 4 patients, an MV-RDOS score of ≥2.6^9^; (7) a decision by the physician in charge of the patient (who was not participating in the study) to adjust suspected suboptimal ventilator settings. Patients were not included in the study if they had any condition making EEG recordings impossible.

### Measurements

#### Dyspnoea

In patients able to communicate with their caregivers, dyspnoea was assessed using a 10 cm left-to-right visual analogue scale (D-VAS) ranging from 0 (complete absence of breathing discomfort) to 10 (maximal imaginable breathing discomfort). In non-communicative patients, the likeliness of being dyspnoeic was assessed using the 5-item MV-RDOS (breathing frequency, inspiratory neck muscle activation, abdominal paradox, heart rate, facial expression of fear; in intubated patients, an MV-RDOS ≥2.6 predicts a dyspnoea-VAS ≥4 with 94% specificity and 57% sensitivity, defining an area under the receiver-operating curve of 95% CI 0.581–0.982)^9^.

#### Respiratory variables

All patients were ventilated with Servo I ventilators (Maquet, Solna, Sweden). Airway opening pressure (Pao) was measured at the distal end of the ventilator circuit with a 0–100 cm H_2_O linear differential pressure transducer (Validyne, Northridge, CA, USA). Flow was measured with single use pneumotachograph (Hamilton Medical AG, Rhazuns, Switzerland) connected in series with the tracheal tube and attached to a 2 cm H_2_O linear differential pressure transducer (DP-45-18; Validyne, Northridge, CA). The signals were digitized at 100 Hz. Tidal volume (Vt), breathing frequency (*f*b) and minute ventilation (V′e) were calculated from the ventilatory flow signal.

#### Electroencephalogram recordings

Electroencephalographic activity was measured with an active electrode system comprising 12 electrodes (9 patients) or 21 electrodes (3 patients) positioned according to the international EEG 10–20 system, referenced to FCz (ActiCap, Brain Products GmbH, Germany). Electrode impedances were kept below 5 kΩ. Signals were amplified and digitalized at a rate of 1000 Hz using a BrainAmp amplifier (Brain Products, GmbH, Germany).

### Experimental sequence

Patients were prepared with the recording devices before the physician in charge (who was not participating in the study) adjusted the ventilator settings. A first 10–15 minute recording of respiratory and neurophysiological signals was performed, at the end of which dyspnoea was re-evaluated (D-VAS or MV-RDOS) (‘PRE’ data). The physician in charge of the patient was then asked to adjust ventilator settings according to his/her own judgment, habits and choices. Then a second 10–15 minute recording of respiratory and neurophysiological signals, followed by dyspnoea evaluation, was performed (‘POST’ data).

### EEG processing

All the computations were performed using Matlab software (Mathworks Inc, USA) version 9.3.

#### Statistical distance between current EEG segment and reference period (classifier)

The detailed methodology of this analysis has been described in previous publications^17,18,21^. In brief, we used an in-house developed and patented algorithm^4^ that classifies brain activity in different conditions using a semi-supervised approach. We tested for modified activity after adjustment of ventilator settings (‘POST’) compared to reference activity before such adjustment (‘PRE’). This involved a learning phase to define reference prototypes (first 20% of the ‘PRE’ period) followed by a detection phase to compare the covariance matrices from the ‘PRE’ and the ‘POST’ periods with the prototypes learned. To perform this analysis, EEG signals from frontal and central channels (F3, Fz, F4, C3, Cz, C4, FP1 and FP2 in 9 patients; F3, Fz, F4, C3, Cz, C4, FP1, FP2, F7, F8, FC3, FC4, FT7 and FT8 in 3 patients) were segmented in 5-second sliding, 50% overlapped windows, down-sampled to 250 Hz and band-pass filtered (8–24 Hz) to enhance motor cortical activity (or mu rhythm^22^) found in this frequency band. Artefactual data windows were removed using an automated method that rejects outlier values on the basis of different statistics (amplitude, linear trend, joint probability and kurtosis)^23,24^. The criterion to reject contaminated epochs was based on z-scores, i.e the difference of a given statistic at a given epoch with respect to the mean across all epochs divided by their standard deviation. Once the reference period was characterized^17,18^ the statistical distance from the reference period was plotted as a function of time and compared for any given EEG segment with a rejection threshold beyond which the EEG covariance becomes statistically different from the reference situation. This is considered indicative of a significant change in brain activity^25^. This threshold is obtained from the distribution of the distances between all the covariance matrices estimated from the reference period, where no significant changes are expected^17,18^.

#### Performance of the classifier

The performance of the classifier was evaluated using a 10-fold cross-validation^18^. The reference period of the ‘PRE’ condition was divided into ten equal parts. Comparison between nine of these parts from the reference period and the data from the ‘POST’ condition was repeated nine times to take into account all combinations. This allowed us to construct Receiver Operating Characteristic curves (ROCs) and calculate the corresponding areas under the curve (AUC) to summarize the sensitivity/specificity ratio of the classifier (one value for each patient; an AUC of 1 indicates perfect discrimination whereas an AUC of 0.5 indicates random discrimination).

#### Visualisation of connectivity

To visualise the changes in dynamical connections evidenced by our classifier, we calculated the EEG’s spatiotemporal dynamics as follows. For each time frame of 5 seconds, we computed an EEG covariance matrix. As non-diagonal elements in covariance matrices express the linear relationship between a pair of channels (a value of 0 indicates that there is no association between a channel pair and a value of 1 that they are identical) we retained only strong connections by applying and arbitrary threshold of 0.9. Relevant connections were represented as lines between the concerned pair of channels in a dynamical topographic image.

#### Pre-inspiratory potentials

This analysis was conducted according to previously described methodology^11,17,26^. In brief, after re-referencing and creating FCz, the EEG signal was segmented using digital trigger pulses automatically generated from threshold crossing of inspiratory airflow. Each of the corresponding segments started 2500 ms before and ended 500 ms after the beginning of inspiration. Segments with a signal gradient in excess of 5 μV/ms or a maximal amplitude in excess of 50 μV for 200 ms or more were automatically rejected. The accepted segments were averaged, and a pre-inspiratory potential was suspected in the presence of a negative deflection preceding inspiration in FCz. In such instances, a linear regression was fitted to the pre-inspiratory data range and a pre-inspiratory potential was considered present if the slope of the corresponding equation was positive and significantly different from zero^17,26^.

### Statistical aspects

Data are summarized as medians and interquartile ranges. Comparisons of values measured before ventilator adjustments (PRE) and after (POST) were conducted using the Wilcoxon matched pairs signed rank test for continuous data and McNemar’s test for dichotomous data. P-values < 0.05 were considered statistically significant.

As this was an initial exploratory study, and in the absence of previous data allowing the proper computation of a sample size, a convenience sample of 12 patients was chosen arbitrarily and considered sufficient to meet the study objectives.

## Results

### Patients

A total of 12 patients (9 men and 3 women, aged 37–87 years) were included in the study (Table 1). At admission, 9 patients were conscious and communicative (Ramsay scale 2 or 3) and 3 patients were non-communicative (Ramsay scale 4). Sedation had been stopped for >24 hours for all patients and for >48 hours for 8 patients.

**Table 1:** Patient characteristics.

Patient	Age	Sex	Indication for MV	SAPSP2	Ramsay	PaO2^a^/PaCO2	DVAS		MV-RDOS		PIPS		Riemann
					scale		Pre	Post	Pre	Post	Pre	Post	AUC
1	65	M	*De novo* acute RF	94	2	137/31	3	0	NA	NA	+	−	0.47
2	50	F	*De novo* acute RF	23	3	245/33.1	4	0	NA	NA	+	+	0.97
3	87	M	Peritonitis	75	2	69.9/29.5	1	1	NA	NA	+	+	0.90
4	74	M	*De novo* ARFacute RF	75	2	67.1/41.5	3	0	NA	NA	+	−	0.94
5	37	F	Acute kidney injury	39	2	69/42	3	1	NA	NA	+	+	0.96
6	76	M	*De novo* acute RF	NK	3	NK	2	0	NA	NA	+	−	1.00
7	62	M	Acute pancreatitis	44	3	78/48	5	1	NA	NA	+	−	0.59
8	48	M	Acute or chronic RF	24	2	85/42	2	0	NA	NA	+	−	0.88
9	50	M	Peritonitis	60	2	70/43	2	0	NA	NA	−	−	0.92
10	78	F	Acute or chronic RF	28	4	NK	NA	NA	2.9	1.9	+	Attenuated	0.70
11	68	M	Sepsis	106	4	NK	NA	NA	2.9	1.8	+	Attenuated	0.54
12	49	M	Acute pancreatitis	47	4	NK	NA	NA	2.7	2.3	+	−	0.36
**Median**				**45**	**2**.**5**		**3**	**0**					**0**.**89**
**IQR**				**38**–**60**	**2**–**3**.**5**		**2**.**5**–**4**	**0**–**1**					**0**.**58**–**0**.**94**

^a^FiO2 35–50%. RF, respiratory failure; IQR, inter-quartile range; SAPSP2, Simplified Acute Physiology Score, Pa, partial pressure; DVAS, dyspnoea visual analogue scale; MV-DOS mechanical ventilation respiratory distress observation scale; PIPS, peak inspiratory potentials; AUC, area under the curve; NK, not known; NA, not applicable.

### Clinical data

During the reference period (before adjustments of ventilator settings [PRE]), median breathing frequency was 24 breaths/min (interquartile range: 20–33) with a median VT of 399 mL (375–478). Median D-VAS was 3.0 (interquartile range 2.5–4.0; minimum-maximum 1–5) in the nine communicative patients, while MV-RDOS was 2.9, 2.9 and 2.7 in the three non-communicative patients.

Ventilator adjustments consisted of increased pressure support (11 cases; +6 [5–8] cm H_2_O), slope adjustments from 0.2–0 s (9 cases), and setting the inspiratory trigger to the most sensitive value (10 cases).

After these ventilator adjustments (POST), breathing frequency was 23 (15–30) (PRE-POST: p = 0.4918) with a VT of 471 (299–634) mL (PRE-POST: p = 0.0093). Median D-VAS was 0.0 (interquartile range 0.0–1.0; minimum-maximum 0–1) in the nine communicative patients (median PRE-POST reduction 3.0 [interquartile range 2–3.5; minimum-maximum 0–4]: p < 0.0078). MV-RDOS was 1.9, 1.8 and 2.3 in the 3 non-communicative patients (PRE-POST reduction of 1, 1.1 and 0.4, respectively). Respiratory discomfort (D-VAS or MV-RDOS) improved after ventilator settings adjustments in 10 of the 12 patients (p = 0.0015).

### EEG data

#### Statistical distance between current EEG segment and reference period (classifier)

**Figure 1:**
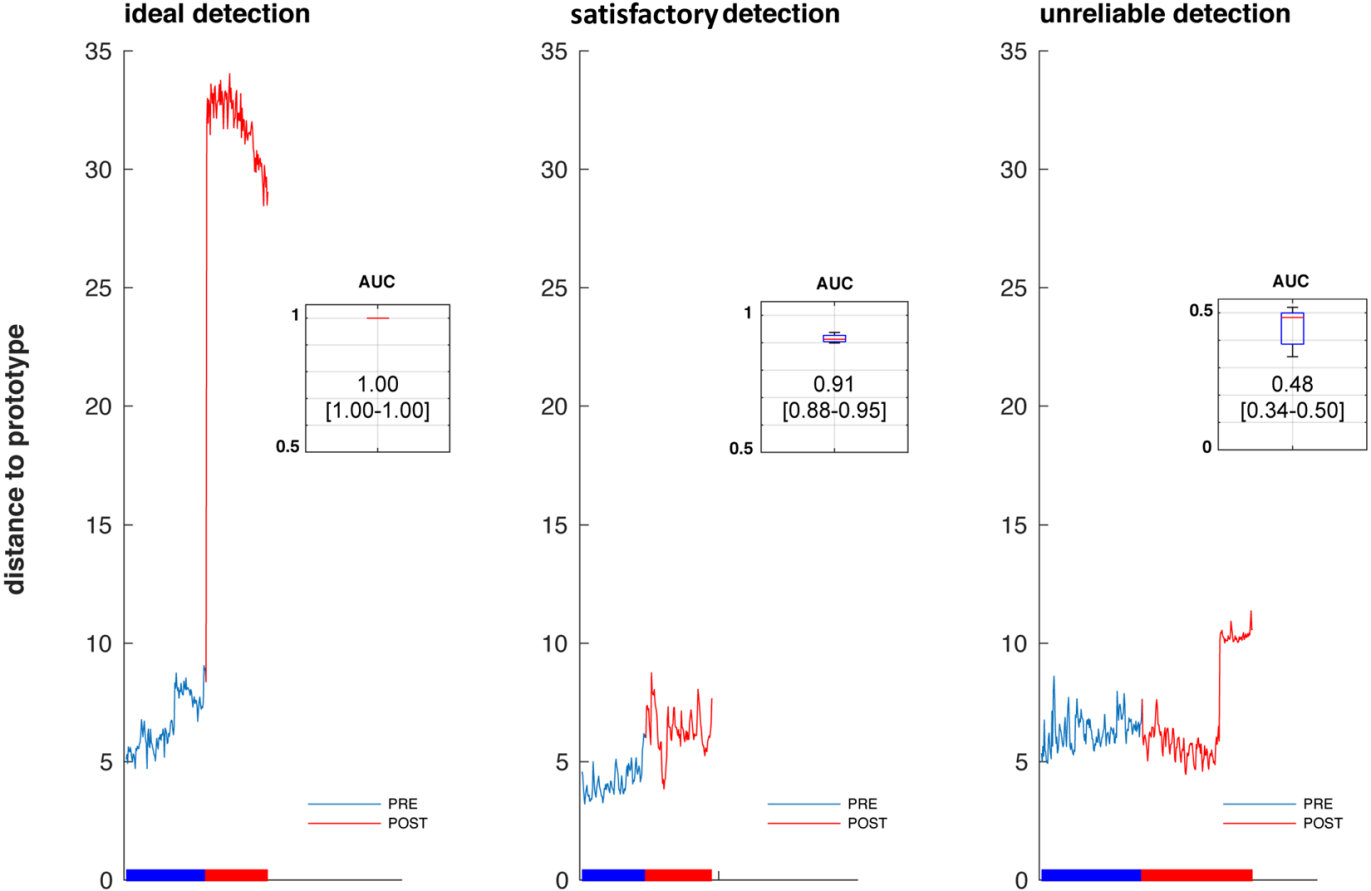
Examples, from left to right, of a case of perfect detection of changes in brain activity following adjustment of ventilator settings (panel A); of a case of imperfect but excellent detection (panel B); and of a case in which no changes were detected (panel C). In each panel, the tracing represents the Riemaniann distance separating the EEG covariance matrix from the matrices representing the reference period (prototypes). The trace is blue when the matrices are considered as belonging to the reference period; it becomes red when the matrices are considered statistically significantly outside of this class. The horizontal bar below the tracing indicates the passage from the ‘PRE’ (before ventilator adjustment) to the ‘POST’ (after ventilator adjustment) condition. On the top right corner of each panel, a cartouche depicts the performance of the classifier to separate the ‘PRE’ and ‘POST’ periods according to the statistical distance, in terms of the corresponding area under the curve (the box delineates the interquartile range of the AUC with indication of its median; the whiskers correspond to the 90^th^ percentile/95^th^ percentile/extreme values).

In all patients, the adjustments of ventilator settings were associated at some point with significant changes in the statistical distance separating current EEG matrices. Classification performance was measured by AUC of the ROC. Median AUC of the sensitivity/specificity ratio of the classifier was 0.89 (interquartile range 0.58–0.94; minimum-maximum 0.36–1). Four patients had AUC <0.6. In the other 8 patients, median AUC was 0.93 (0.90–0.96). Figure 1 shows examples of (A) perfect detection of the EEG changes following adjustment of ventilator settings, (B) satisfactory detection, and (C) AUC too low to consider the detection reliable.

#### Visualization of connectivity

**Figure 2:**
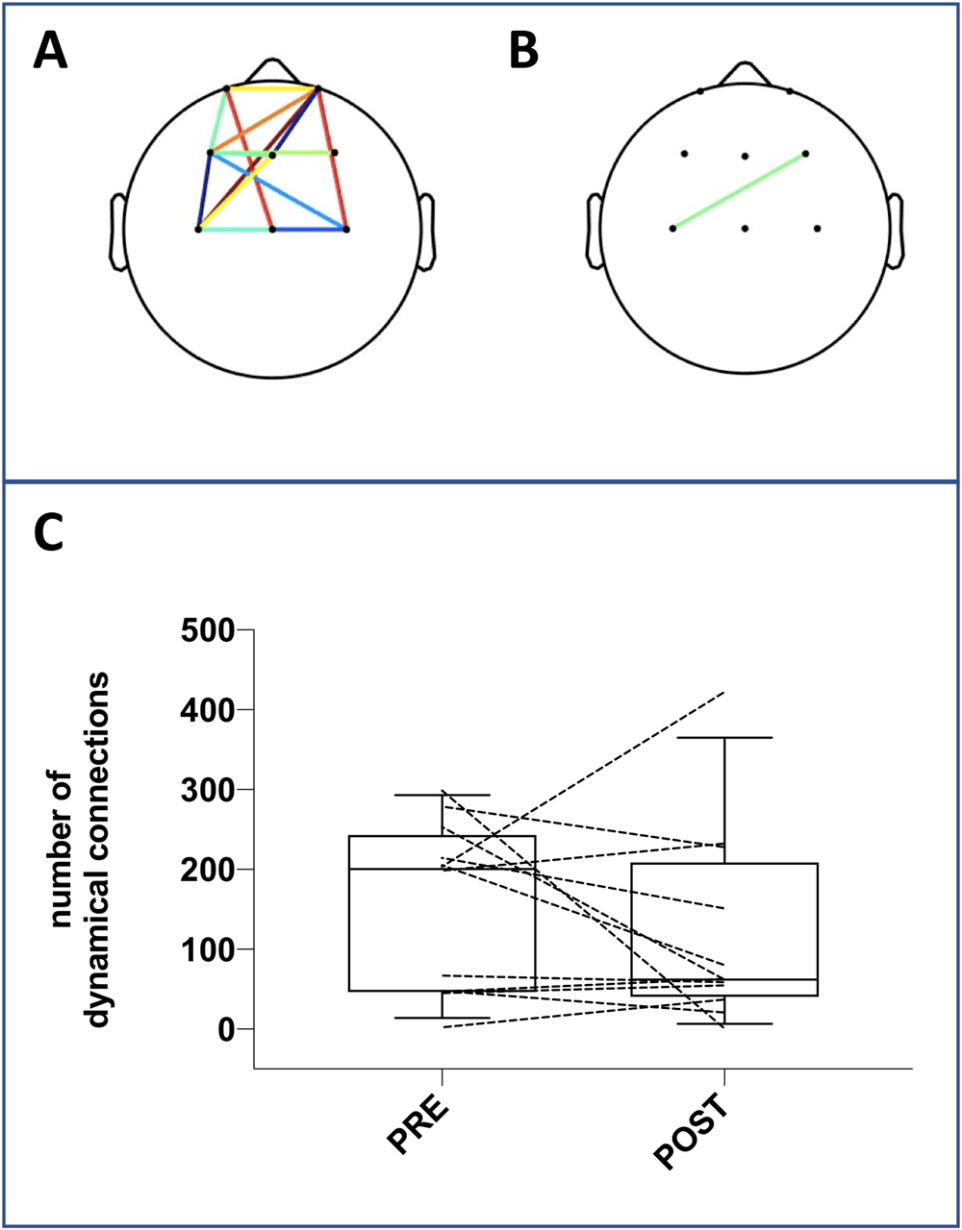
Connections between pairs of channels before the adjustments of ventilator settings (**A**) and after these adjustments (**B**) in a patient (#6) in whom the performance of the Riemannian classifier yielded an AUC of 1 (same patient as in Fig. 1 panel A). See the corresponding video file provided in electronic supplement. (Panel C) Shows the total number of connections during each recording period (before and after adjustments of ventilator settings) in the overall population (the boxes delineate the interquartile range with indication of the median; the whiskers correspond to the 90^th^ percentiles of the distribution; dotted lines correspond to individual patients).

The spatiotemporal dynamics analysis showed that in some subjects the change in statistical distance following ventilator settings adjustments was associated with a dramatic decrease in the number of dynamical connections (reduction by more than 50% of the number of connections in 5 subjects, see Supplementary Video). There was, however, no systematic pattern and overall the decrease in the number of connections did not reach statistical significance (p = 0.09) (Fig. 2).

#### Pre-inspiratory potentials

**Figure 3:**
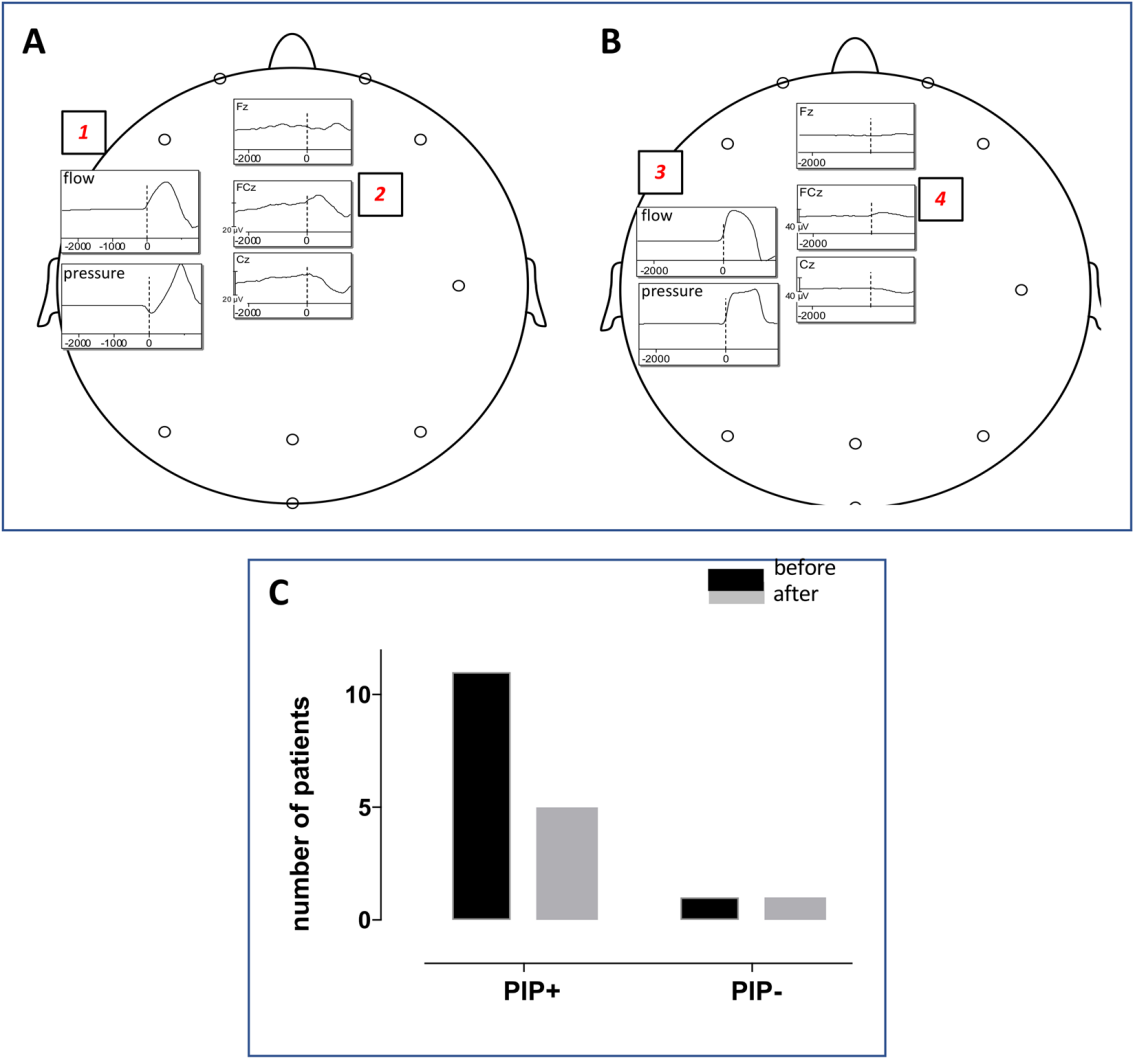
(Panel A) Example, in one patient, of flow and pressure traces recorded before adjustment of ventilator settings (square #1). The corresponding FCz and Cz inspiratory-locked segments average show a negative deflection preceding inspiration (square #2), namely a pre-inspiratory potential (PIP). (Panel B) Flow and pressure traces recorded after adjustment of ventilator settings (square #3). The FCz and Cz traces show the complete disappearance of the previously recorded pre-inspiratory potential. (Panel C) Among the 11 patients who exhibited a PIP before adjustment of ventilator settings, 4 did so after this adjustment. The patient who did not exhibit any PIP before still did not do so after adjustment.

Before adjustment of ventilator settings, 11 patients exhibited pre-inspiratory potentials, including the 3 non-communicative patients. After adjustment of the ventilator settings the pre-inspiratory potentials disappeared or almost disappeared in 7 cases (p = 0.0338) (Fig. 3). In 5 of these cases, the PIPs could no longer be identified (complete disappearance, i.e. a flat pre-inspiratory EEG). In the two other cases, a PIP was still detectable but the departure of the EEG signal from baseline was sufficiently small as to leave a doubt on the existence of the potential (extreme attenuation). The one patient with no pre-inspiratory potential before ventilator adjustments remained the same after these adjustments.

#### Consistency between clinical and EEG data

**Figure 4:**
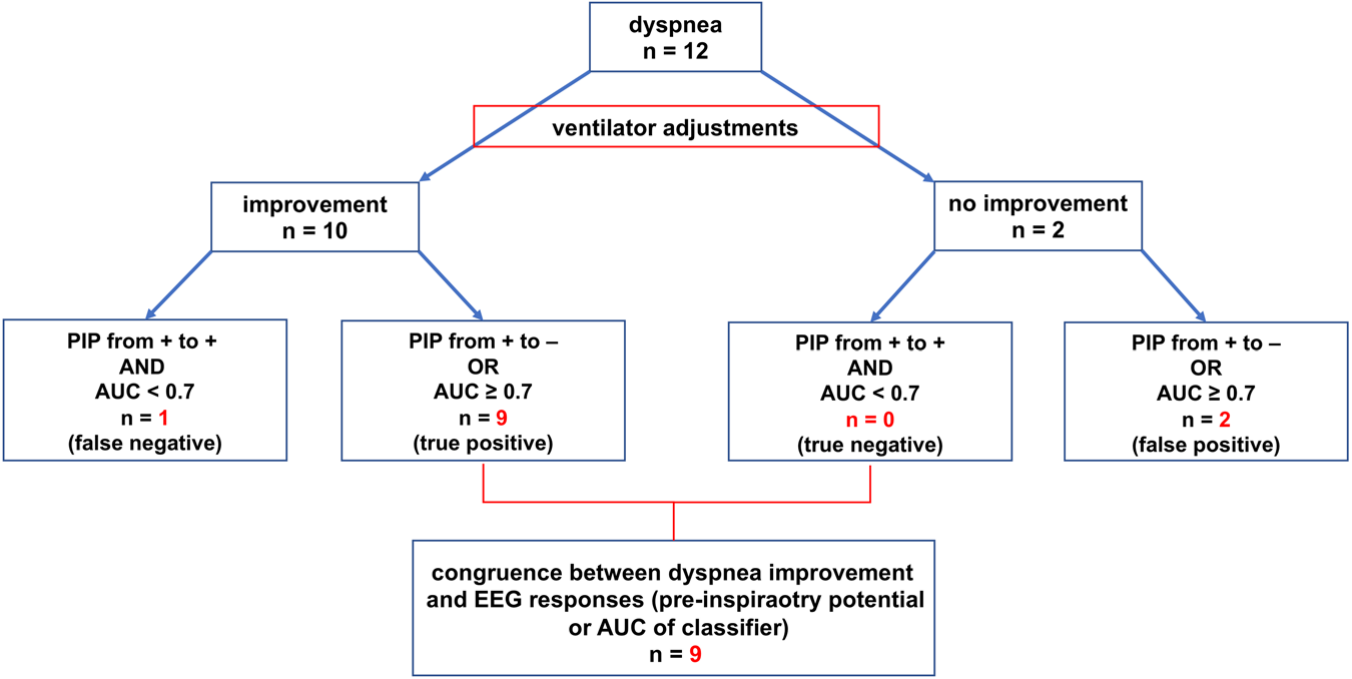
Congruence between dyspnoea improvement following adjustments of ventilator settings and detection of brain activity changes through combined analysis of the EEG classifier area under the curve (AUC) and of pre-inspiratory potentials (PIP).

Figure 4 illustrates the congruence between dyspnoea improvement following adjustments of ventilator settings and detection of brain activity changes through combined analysis of the EEG classifier performances (AUC) and PIPs.

## Discussion

This study shows that modifying ventilator settings in MV patients can induce changes in brain activity that can be detected using scalp EEG electrodes. In our patients, with confirmed or suspected dyspnoea, the observed EEG changes were generally consistent with clinical improvements following ventilator adjustments. To our knowledge, this study is the first to indicate that dyspnoea changes can be associated with measurable EEG changes in patients receiving MV during an ICU stay. Consistent with observations made in patients with chronic respiratory failure^12^, the present study confirms that PIPs can be useful in detecting respiratory-related cortical activation and deactivation in a clinical context. Additionally, we were able to distinguish the states before and after ventilator adjustments using algorithms designed to evaluate cortical connectivity continuously and in real-time and validated during experimental inspiratory loading in humans^17^.

Several physiological pathways can link MV to brain activity, including hypoxia^27^, hypercapnia^28^, and the afferent neural traffic generated by breathing movements. Respiratory-related signatures can be detected in the EEG in relationship with natural breathing^29–31^, voluntary respiratory movements^32^, or acute changes in upper airway resistance (respiratory-related evoked potentials)^33,34^. Furthermore, respiratory loading has been shown to be associated with pre-inspiratory or pre-expiratory cortical potentials^11,12,17,26^, and CO_2_ rebreathing has been associated with yet another type of EEG signature^16^. Regarding MV, subjecting normal individuals to positive pressure breathing while preventing hypocapnia decreases neural inspiratory output^35^ and depresses the electromyographic response of the diaphragm to transcranial magnetic stimulation^36^. The adjustments of ventilator settings to relieve dyspnoea in patients included in the present study consisted of increased pressure support, faster delivery of the pressure support, and improved trigger sensitivity, i.e. a combination of increased assistance and decreased loading. This resulted in increased tidal volume, known to powerfully relieve dyspnoea^37,38^ with a corresponding relief of anxiety^1^. It also resulted in increased ventilation. We did not monitor end-tidal CO_2_ and can therefore not ascertain to what extent this modified blood gases. However, because our patients were not hypercapnic to begin with (in fact, most were hypocapnic), their improved dyspnoea was unlikely to have been driven by a reduction in CO_2_ (of note, pulsed transcutaneous oxygen saturation was continuously monitored, without discernible changes during the procedure). Carbon dioxide changes could, however, have had an impact on any CO_2_-related EEG activity^16^. It is therefore not surprising that we were able to detect EEG changes in response to the changes in breathing regimen that followed ventilator settings adjustments. The nature of the EEG analyses that we conducted suggests these EEG changes to be related to cortical connectivity^17,18^. Cortical connectivity intrinsically underlies premotor potentials in general, including PIPs as their respiratory variant. Whether it also underlies CO_2_-related EEG activities^16^ is not known.

**Figure 5:**
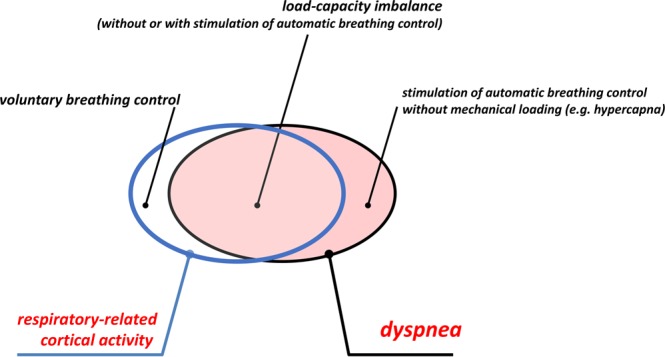
Theoretical framework of the relationship between respiratory-related cortical activity (blue frame) and dyspnoea (black frame), the importance of the overlap between the two being unknown.

The two EEG indicators that we used to characterize breathing differ in nature. Pre-inspiratory potentials denote a motor activity, are specific to breathing, and are indirectly and contextually related to breathlessness. They can be observed in the absence of dyspnoea during voluntary ventilatory manoeuvres^17,39,40^ or in patients with defective automatic breathing control^40^. Given their absence during normal natural breathing^11,39^, their presence in patients in whom the *a priori* probability of being dyspnoeic is high should trigger a targeted clinical enquiry. All our patients exhibited PIPs before ventilator adjustments, which strongly suggests that they were exposed to a mechanical inspiratory loading in spite of mechanical ventilation. PIPs disappeared subsequently or were attenuated in a statistically significant number of cases. This provides an additional argument in support of the link between PIPs and dyspnoea. However, PIPs can be absent despite dyspnoea if the main driver is hypercapnia rather than excessive mechanical load^11^ (Fig. 5), but their identification and monitoring is technically demanding and cannot be done in real-time. In contrast, the statistical classifiers that we used to characterize brain states offer the possibility of real-time PIP detection^18,19^ and are considered highly promising in the context of brain-computer interface development^19^. They would react to EEG changes related to both respiratory loading and carbon dioxide^16^: importantly, this helps to explain some of our observations of improved dyspnoea congruent with the detection of EEG changes by the Riemaniann approach in spite of the persistence of PIPs. However, these statistical classifiers are not specific to the detection of breathing-related EEG changes. In our study, changes in brain state were detected immediately after ventilator adjustments that resulted in improved breathing comfort. It is therefore reasonable to hypothesize that they were respiratory-driven. However, in a real-life configuration, the lack of respiratory specificity would be a major issue with a risk of frequent false respiratory alarms. Future studies are needed to determine the best way to answer this question. One possible solution would be to use the Riemannian detector to trigger a PIP analysis, although breathlessness is possible in the absence of PIPs, and PIPs can persist despite an improvement in dyspnoea. In our patients the congruence between the changes in statistical detection and PIP analysis was not excellent. This raises the question of the level of respiratory discomfort that can be tolerated during MV or if any such discomfort can be tolerated at all. Another possible solution to make a Riemaniann EEG classifier specific to respiration would be to include respiratory data in the analysis. This could involve a combined analysis of the EEG, a respiratory electromyogram^4^, and/or a flow or flow-derived signal.

This was a proof-of-concept study with several limitations. It was conducted in a small and selected number of patients and in a very specialized environment rather than in routine conditions. Extrapolation of the results to the wider population is therefore not possible at this stage. Importantly, the performance of current and/or future EEG algorithms to identify occult respiratory suffering in MV patients in real-life depends on many factors, including the degree of patient consciousness, drug interference, and the development of EEG headsets that are acceptable for both patients and caregivers. All these issues need further evaluation.

**Figure 6:**
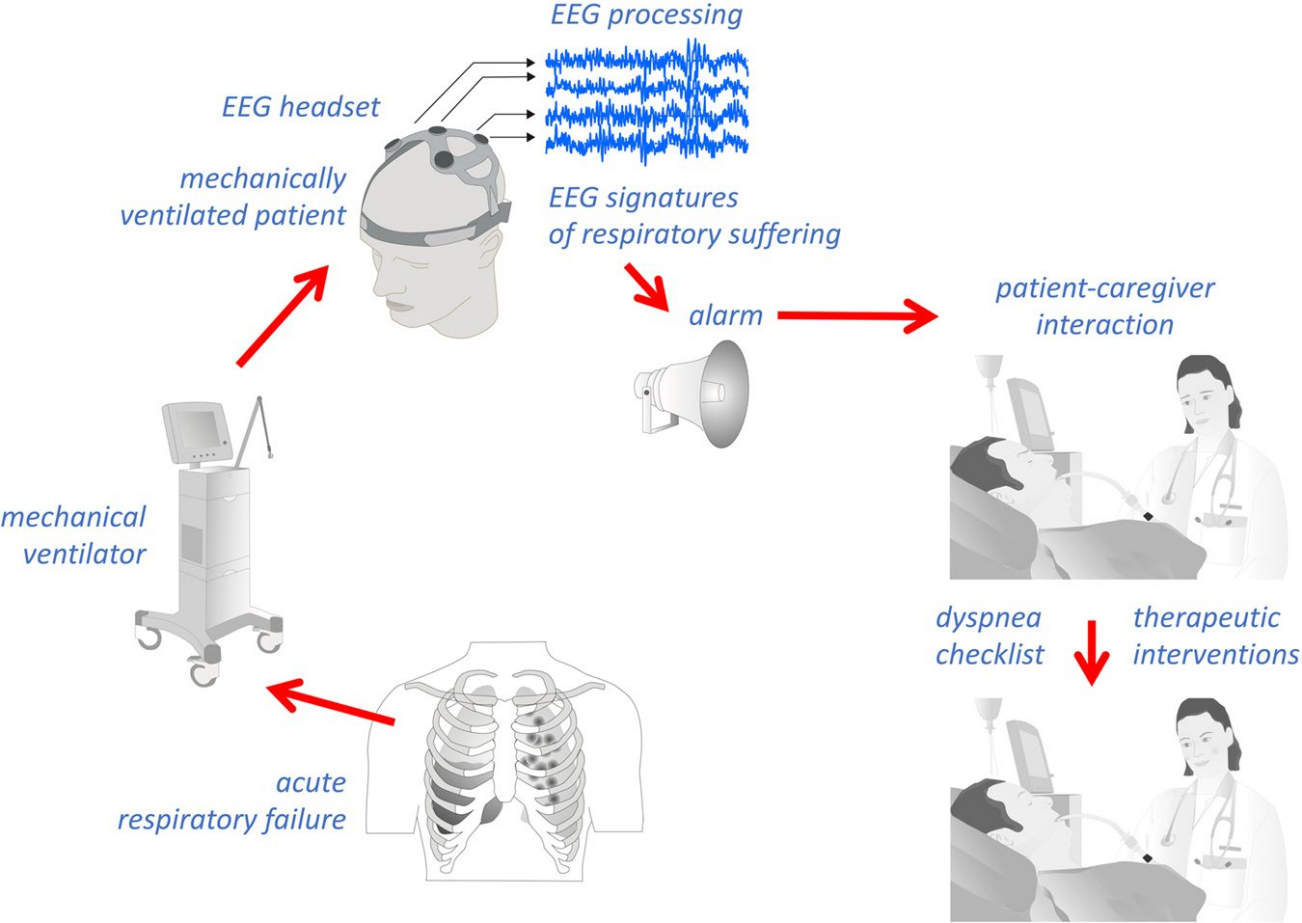
Theoretical framework of the putative role of EEG respiratory neuromarkers in the management of dyspnoea in patients receiving mechanical ventilation.

In conclusion, our results show that adjusting ventilator settings to relieve dyspnoea produced detectable changes in brain activity, justifying future research in this area. The general aim of such research will be to increase the number of patient-caregiver interactions pertaining to breathing comfort, through an EEG alarm allowing caregivers to launch diagnostic and therapeutic procedures (Fig. 6) and to determine the impact on clinical outcomes in critically ill MV patients.

## Supplementary information

Dynamic connectivity and ventilator settings

## Data Availability

Recordings can be made available to all interested researchers on simple request from the Corresponding Author.

## References

[1] Schmidt M (2011). Dyspnea in mechanically ventilated critically ill patients. Crit Care Med.

[2] Chanques G, Nelson J, Puntillo K (2015). Five patient symptoms that you should evaluate every day. Intensive Care Med.

[3] Decavele M, Similowski T, Demoule A (2019). Detection and management of dyspnea in mechanically ventilated patients. Curr Opin Crit Care.

[4] Schmidt M (2014). Unrecognized suffering in the ICU: addressing dyspnea in mechanically ventilated patients. Intensive Care Med.

[5] Binks AP, Desjardin S, Riker R (2017). ICU clinicians underestimate breathing discomfort in ventilated subjects. Respir Care.

[6] Gentzler Eliza R., Derry Heather, Ouyang Daniel J., Lief Lindsay, Berlin David A., Xu Cici Jiehui, Maciejewski Paul K., Prigerson Holly G. (2019). Underdetection and Undertreatment of Dyspnea in Critically Ill Patients. American Journal of Respiratory and Critical Care Medicine.

[7] Haugdahl HS (2015). Underestimation of patient breathlessness by nurses and physicians during a spontaneous breathing trial. Am J Respir Crit Care Med.

[8] Campbell ML, Templin T, Walch J (2010). A Respiratory Distress Observation Scale for patients unable to self-report dyspnea. J Palliat Med.

[9] Decavele M (2018). Management of dyspnea in the noncommunicative patients: consider hetero-evaluation scales. Chest.

[10] Persichini R (2015). Diagnostic Accuracy of Respiratory Distress Observation Scales as surrogates of dyspnea self-report in Intensive Care Unit patients. Anesthesiology.

[11] Raux M (2007). Cerebral cortex activation during experimentally induced ventilator fighting in normal humans receiving noninvasive mechanical ventilation. Anesthesiology.

[12] Georges M (2016). Cortical drive to breathe in amyotrophic lateral sclerosis: a dyspnoea-worsening defence?. Eur Respir J.

[13] Nierat Marie-Cécile, Raux M, Redolfi S, Gonzalez-Bermejo J, Biondi G, Straus C, Rivals I, Morélot-Panzini C, Similowski T (2018). Neuroergonomic and psychometric evaluation of full-face crew oxygen masks respiratory tolerance: a proof-of-concept study. Journal of the Royal Army Medical Corps.

[14] Banzett RB (2000). Breathlessness in humans activates insular cortex. Neuroreport.

[15] Binks AP, Evans KC, Reed JD, Moosavi SH, Banzett RB (2014). The time-course of cortico-limbic neural responses to air hunger. Respir Physiol Neurobiol.

[16] Seino T, Masaoka Y, Inagaki K, Izumizaki M (2015). Breathlessness-related brain activation: Electroencephalogram Dipole Modeling Analysis. Showa Univ J Med Sci.

[17] Hudson AL (2016). Electroencephalographic detection of respiratory-related cortical activity in humans: from event-related approaches to continuous connectivity evaluation. J Neurophysiol.

[18] Navarro-Sune X (2017). Riemannian geometry applied to detection of respiratory states from EEG signals: the basis for a brain-ventilator interface. IEEE Trans Biomed Eng.

[19] Yger F, Berar M, Lotte F (2017). Riemannian approaches in brain-computer interfaces: a review. IEEE Transactions on Neural Systems and Rehabilitation Engineering.

[20] Ramsay M, Savege T, Simpson B, Goodwin R (1974). Controlled sedation with alphaxalone-alphadolone. Br Med J..

[21] Grosselin F, Navarro-Sune X, Raux M, Similowski T, Chavez M (2018). CARE-rCortex: a Matlab toolbox for the analysis of CArdio-REspiratory-related activity in the cortex. J Neurosci Methods.

[22] Pfurtscheller G, Lopes da Silva FH (1999). Event-related EEG/MEG synchronization and desynchronization: basic principles. Clin Neurophysiol.

[23] Delorme A (2011). EEGLAB, SIFT, NFT, BCILAB, and ERICA: new tools for advanced EEG processing. Comput Intell Neurosci.

[24] Delorme A, Sejnowski T, Makeig S (2007). Enhanced detection of artifacts in EEG data using higher-order statistics and independent component analysis. Neuroimage.

[25] Mason SJ, Graham NE (2002). Areas beneath the relative operating characteristics (ROC) and relative operating levels (ROL) curves: statistical significance and interpretation. Quarterly Journal of the Royal Meteorological Society.

[26] Raux M, Tremoureux L, Couturier A, Hug F, Similowski T (2010). Simplified recording technique for the identification of inspiratory premotor potentials in humans. Respir Physiol Neurobiol.

[27] Ikeda T, Yamada S, Imada T, Matsuda H, Kazama T (2009). Influence of hypobaric hypoxia on bispectral index and spectral entropy in volunteers. Acta Anaesthesiol Scand.

[28] Corfield DR, McKay LC (2016). Regional Cerebrovascular responses to hypercapnia and hypoxia. Adv Exp Med Biol.

[29] Grözinger B, Kriebel J, Kornhuber H (1974). Respiration correlated brain potentials. Journal of Interdisiplinary Cycle Research.

[30] Busek P, Kemlink D (2005). The influence of the respiratory cycle on the EEG. Physiol Res.

[31] Heck DH (2016). Breathing as a fundamental rhythm of brain function. Front Neural Circuits.

[32] Herrero JL, Khuvis S, Yeagle E, Cerf M, Mehta AD (2018). Breathing above the brain stem: volitional control and attentional modulation in humans. J Neurophysiol.

[33] Davenport PW, Friedman WA, Thompson FJ, Franzen O (1986). Respiratory-related cortical potentials evoked by inspiratory occlusion in humans. J Appl Physiol (1985).

[34] Knafelc M, Davenport PW (1997). Relationship between resistive loads and P1 peak of respiratory-related evoked potential. J Appl Physiol (1985).

[35] Fauroux B (1998). Nonchemical influence of inspiratory pressure support on inspiratory activity in humans. J Appl Physiol (1985).

[36] Sharshar T (2004). Depression of diaphragm motor cortex excitability during mechanical ventilation. J Appl Physiol (1985).

[37] Bloch-Salisbury E, Spengler CM, Brown R, Banzett RB (1998). Self-control and external control of mechanical ventilation give equal air hunger relief. Am J Respir Crit Care Med.

[38] Manning HL (1992). Reduced tidal volume increases ‘air hunger’ at fixed PCO2 in ventilated quadriplegics. Respir Physiol.

[39] Macefield G, Gandevia SC (1991). The cortical drive to human respiratory muscles in the awake state assessed by premotor cerebral potentials. J Physiol.

[40] Tremoureux L (2014). Does the supplementary motor area keep patients with Ondine’s curse syndrome breathing while awake?. PLoS One.

